# Pennsylvania's Transition to Medicaid Managed Long-Term Services and Supports

**DOI:** 10.1093/geroni/igab046.1751

**Published:** 2021-12-17

**Authors:** Howard Degenholtz

**Affiliations:** University of Pennsylvaniz, Pittsburgh, Pennsylvania, United States

## Abstract

Implemented through five health plans, Ohio’s MyCare demonstration began in 2014 and was designed to coordinate primary, acute care, behavioral health and long-term services in the major urban areas of the state. Individuals who are dually eligible for both Medicaid and Medicare and who reside in specified geographic regions must enroll into a managed MyCare plan. MyCare beneficiaries are assigned to two primary categories: community well and those needing long-term services and supports (LTSS). Individuals receiving the integrated MyCare intervention were expected to have lower acute care hospitalizations, lower long-term nursing home use, better longevity and lower overall health and long-term care costs. Using a propensity score matching design, the evaluation compared MyCare enrollees to comparison group members in non-MyCare counties of the state, using Medicaid and Medicare claims data. The 120,000 MyCare program participants represented about half of the dual eligible individuals in the state.

